# Etanercept leads to a rapid recovery of a Dabrafenib‐/Trametinib‐associated toxic epidermal necrolysis‐like severe skin reaction

**DOI:** 10.1002/ski2.185

**Published:** 2022-11-05

**Authors:** Krista Yordanova, Claudia Pföhler, Luca F. Schweitzer, Catherine Bourg, Leonie Adam, Thomas Vogt

**Affiliations:** ^1^ Department of Dermatology, Venereology and Allergology Saarland University Medical Center Homburg/Saar Germany

## Abstract

Targeted therapy with BRAF‐ and MEK‐Inhibitors (BRAFi, MEKi) provides an excellent therapeutic option for patients with malignant melanomas with a BRAF‐Mutation. Mild cutaneous adverse events have been common under the BRAF‐ and MEK‐Inhibitor therapy, on the contrary, severe cutaneous adverse reactions to drugs (SCARs) are rarely reported. We present the case of a 59‐ year‐old female patient who after the resection of cutaneous in‐transit metastases of a malignant melanoma received one adjuvant cycle of Nivolumab followed by a switch of the therapy to an oral BRAFi/MEKi therapy. 3–4 Weeks after the therapy switch she developed high fever, chills, progredient general weakness, headaches, abdominal complaints, generalised rash as well as thrombocytopaenia, eosinophilia, elevated liver enzymes, declining kidney, and pulmonary function as well as a maculopapular exanthema. She was diagnosed with drug reaction with eosinophilia and systemic symptoms (DRESS) and quickly started recovery after initiation of a high steroid substitution. Under steroid dose reduction, the exanthema worsened and toxic epidermal necrolysis (TEN) was histologically diagnosed. After a series of unsuccessful therapeutic approaches (high dose steroid, human immunoglobulins and ciclosporin) the patient received a single dose of the TNF‐alpha inhibitor etanercept, which led to a quick recovery. This case demonstrates that DRESS and TEN can present a spectrum of possibly transitioning SCARs providing a diagnostic and therapeutic challenge. Nevertheless, in a such complicated therapeutic setting, etanercept may be lifesaving even after multiple previous unsuccessful therapies. This effective approach provides evidence SCARs due to BRAF/MEK targeted therapy may be driven by TNF‐alpha.

## Graphical abstract

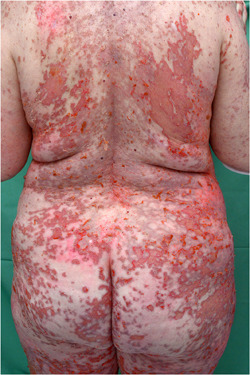

We present a case of a dabrafenib and trametinib induced drug reaction with eosinophilia and systemic symptoms‐like syndrome, that progresses to a toxic epidermal necrolysis‐like severe skin reaction. The condition was resistant to conventional immune‐suppressive therapy with high‐dose steroids, ciclosporin, or human immunoglobulin. The only effective therapy approach was a single application of the TNF‐alpha inhibitor etanercept 50 mg subcutaneously.

## 1 INTRODUCTION

Targeted therapy with BRAF‐ and MEK‐Inhibitors (BRAFi and MEKi) provides an excellent therapeutic option for patients with malignant melanomas harbouring a BRAF‐Mutation.^1^ The combination of BRAFi and MEKi has become a standard of care and is approved for the treatment of advanced melanoma and as an adjuvant treatment in stage III.^2^ Mild cutaneous adverse events such as rash and pruritus are common under the BRAFi/MEKi therapy.^3^ Severe cutaneous adverse reactions to drugs (SCARs) are rarely reported. These include acute generalised pustulosis (AGEP), drug reaction with eosinophilia and systemic symptoms (DRESS) and Stevens‐Johnson syndrome‐toxic epidermal necrolysis (SJS‐TEN).^4^, ^5^ These SCARs vary in their clinical manifestation, severity, and prognosis and can manifest as a life‐threatening disease. The deciding factor for the diagnosis is the morphology of the individual lesions, the distribution pattern as well as the general symptoms.^6^ As DRESS presents systemic complications, TEN presents epidermal necrolysis that can vary in the degrees of severity. We present a case of SCAR under therapy with dabrafenib and trametinib that initially fulfiled the criteria of DRESS and finally progressed to TEN. Prompt remission could be achieved by a single dose of the TNF‐alpha inhibitor etanercept after the failure of conventional immunosuppressive drugs and immunoglobulins.

## 2 CASE REPORT

**FIGURE 1:**
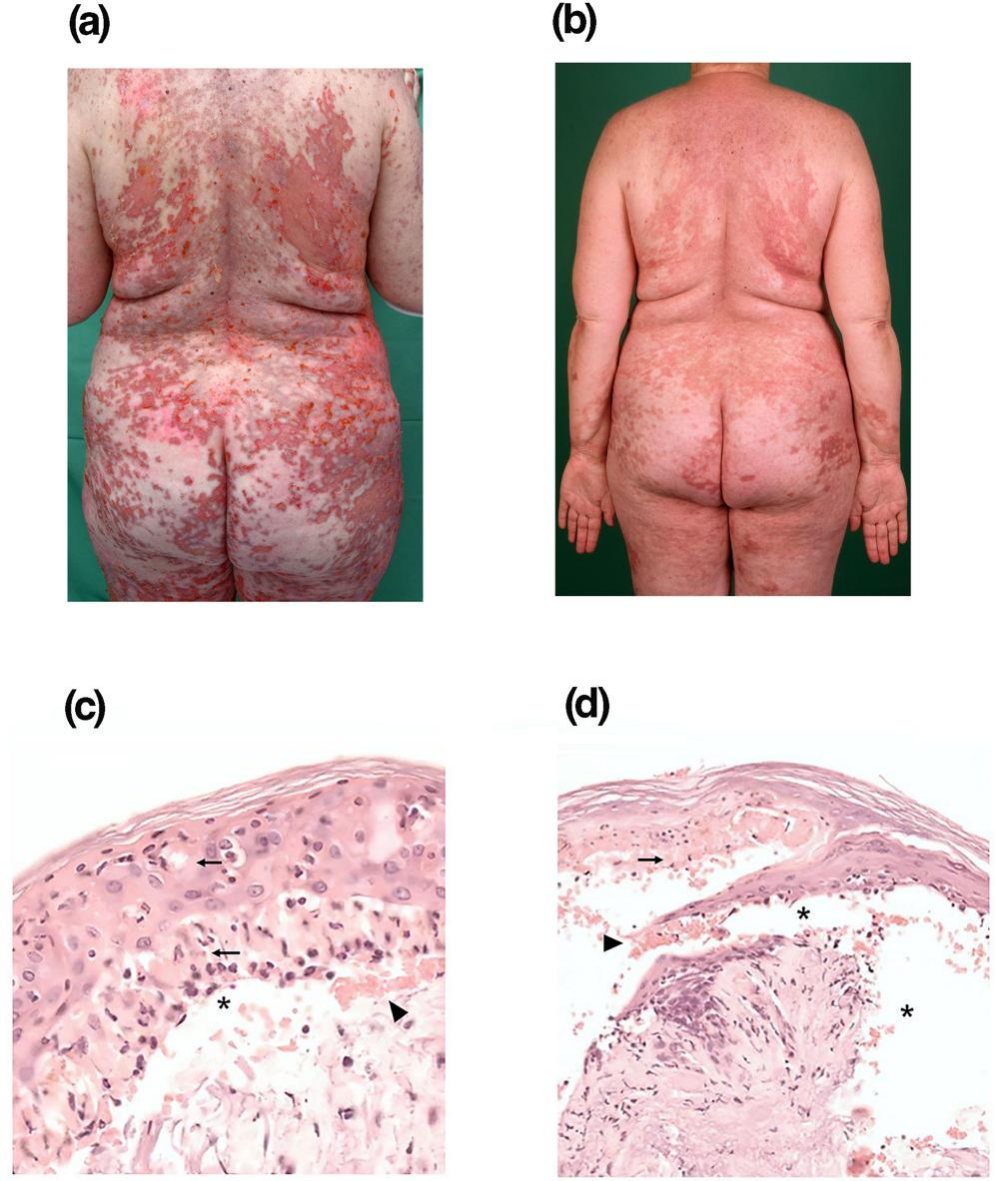
(a) maculopapular exanthem with erythema exsudativum multiforme‐like (EEM)‐like scaling of the skin; (b) Rash reepithelisation of the skin after a single shot of the TNF‐alpha blocker etanercept 50 mg. (c, d) Punch‐biopsy shows masses of apoptotic keratinocytes (arrows), erythrocytes (arrowhead) and a gap area (asterisk) representing a beginning detachment of the epidermis, confirming the development of the drug reaction with eosinophilia and systemic symptoms (DRESS) condition into toxic epidermal necrolysis (TEN); haematoxylin and eosin. Magnification, ×400 (a), ×200 (b)

We report the case of a 59‐year‐old female patient, who received one cycle of Nivolumab 480 mg intravenously in an adjuvant setting after surgical resection of cutaneous in‐transit metastases. At the time, the mutation status of the tumour was unknown. After detection of a BRAF pV600E mutation, the adjuvant therapy was switched, by request of the patient, to an oral targeted therapy with Dabrafenib and Trametinib. 3–4 weeks after initiation of this therapy the patient developed fever (up to 40°C), chills, headaches, abdominal complaints, generalised rash and progredient weakness. The targeted therapy was discontinued and the patient was urgently hospitalised. Lab results showed an elevated c‐reactive protein 30.7 mg/dl, thrombocytopaenia 23 000/μL, lymphopenia 550/μL, eosinophilia 9%, elevated liver enzymes and declined kidney function. The pulmonary function and general condition of the patient were rapidly worsening. The macular rash converted into a maculopapular exanthema. A CT scan showed atypical infiltrations in both lungs. Under the suspicion of pneumonia and sepsis, antibiotic therapy with ampicillin plus ceftriaxone was initiated. A COVID19 Infection was repeatedly excluded. As the general condition of the patient worsened despite the antibiotic therapy, non‐invasive ventilation had to be initiated. Considering the course of the symptoms and the absence of improvement under conventional therapy, a severe drug reaction was considered. Hence, DRESS resulting from BRAFi/MEKi therapy was diagnosed and therapy with 2 mg/kg methylprednisolone/day was initiated. Subsequently, the patient showed a quick recovery of her pulmonary function, the lab results normalised and the exanthema began to fade. The patient was discharged with an orally administrated dose of 40 mg prednisolone/day. Two days later, she experienced progredient itching, worsening of the exanthema and we saw a maculopapular exanthema with an erythema exsudativum multiforme‐like (EEM) scaling of the skin of the trunk, upper arms and upper legs (Figure 1a). The Nikolsky's sign was positive. The patient was hospitalised again and received 3 mg/kg/day of methylprednisolone intravenously. Despite this medication, the cutaneous symptoms worsened and she developed an erythroderma and progressive epidermal necrosis of the skin. A punch‐biopsy confirmed masses of apoptotic keratinocytes and beginning TEN (Figure 1c,d), so we initiated therapy with high dose immunoglobulin 0.5 g/kg over 4 days and ciclosporin 200 mg daily. Nevertheless, the skin condition deteriorated rapidly. All previous medications were then discontinued and a single shot of the TNF‐alpha blocker etanercept 50 mg was applied subcutaneously. Just 24 h later the progress of the epidermal necroses cease and within a few days, we observed a rapid consecutive reepithelisation. The patient was discharged a week later (Figure 1b).

## 3 DISCUSSION

The cutaneous side effects of the targeted therapy with BRAFi/MEKi range from a mild exanthema through exanthemas with systemic reactions and may reach the severity of SJS/TEN in rare cases. Commonly, cutaneous reactions are mild and develop at the beginning of the therapy.^3^ SCARs such as DRESS and TEN/SJS are more uncommon. Previous treatment with an anti‐PD1 drug, as in the case reported here, may be associated with an early onset of the symptoms and heightened severity.^7^

We also cannot fully exclude that antibiotic therapy may have contributed to the worsening of the condition and the progression in to a TEN like Syndrome, as the antibiotic therapy is a known triggering factor of a TEN. On the other hand, us the condition already developed before the beginning of an antibiotic therapy, and again worsened after a reduction of steroids, the most plausible reason was the BRAF/MEK Inhibition.

Overlapping cases displaying clinical features and fulfiling the criteria of both DRESS and TEN are possible.^8^ In the present case, we first observed a DRESS‐like condition, that developed into a TEN‐like Syndrome. Initially, the patient displayed fever, chills and exanthema. The lab results showed haematological changes, elevated liver enzymes and declining kidney function, fulfiling the RegiSCAR criteria of a DRESS syndrome.^9^ In the further course, after initial improvement under a high steroid dose therapy, while tapering down the steroids, the exanthema progressed into an erythroderma with exfoliative dermatitis presenting a histological picture of TEN‐Syndrome.

Although the pathophysiology of SCARs is not completely understood, both DRESS and TEN are classified as a type IV, delayed hypersensitivity reaction.^8^ Drug reactive T‐cells recruit and activate immune cells, leading to an inflammatory skin reaction and extensive tissue damage.^4^, ^10^ Administration of high doses of corticosteroids is still considered to be the therapy of choice.^5^ In cases of steroid resistance, further therapeutic options such as high‐dose immunoglobulin^11^ can be considered, but to date, this is still a matter of debate. TNF‐alpha inhibitors like infliximab and etanercept are effective treatment options for SCARs.^5^, ^12^, ^13^ Etanercept is a chimaeric monoclonal TNF‐alpha antibody, used in the therapy of autoimmune diseases such as rheumatoid arthritis or plaque psoriasis.^14^ Patients with TEN show a significant improvement of reepithelialisation after Etanercept application compared with traditional steroid‐therapy.^15^ *Previous case reports as well as a randomised controlled clinical trial showed a significant improvement of reepitalisation in patients with TEN after Etanercept application in comparison with traditional therapy with corticosteroids* (*Wang et al, 2018*) TNF‐alpha inhibition reduces proinflammatory cytokines and the secretion of granulysin.^15^, ^16^ As elevated levels of TNF‐alpha, chemokines and cytokines have been detected at the onset of DRESS Syndrome as well as in TEN‐patients treatment with a TNF‐alpha antibody seems plausible for both.^4^, ^12^, ^13^, ^17^ A HHV6, HHV7, CMV and EBV virus reactivation has previously been described in patients with DRESS Syndrome. A screening prior to the beginning of the TNF‐alpha inhibitor therapy should be strongly considered, as the initiation of during an active virus infection could be fatal for the patent.^18^ In our case, after an unsuccessful “classic” therapeutical approach a single dose of etanercept led to rapid initialisation of reepithelialisation.

The development of severe adverse events under the dabrafenib/trametinib therapy permanently contraindicates its further use. This can significantly limit the therapeutic options for patients with metastatic melanoma. It is possible to successfully switch to another BRAFi/MEKi representative after a severe adverse event.^19^ Fortunately, our patient was undergoing adjuvant BRAFi/MEKi therapy at the time. As she was tumour‐free, she received no further adjuvant therapy after the discontinuation of dabrafenib and trametinib.

In conclusion, we provide first evidence that SCARs due to BRAFi/MEKi targeted therapy may be driven by TNF‐alpha and therefore be responsive to etanercept. The clinical improvement in our patient was dramatic and possibly lifesaving. Our observation may help clinicians in comparable situations and spark further studies in this direction. Furthermore, our case demonstrates that DRESS and TEN may represent a spectrum of related and possibly merging SCARs as sequelae of BRAF/MEK inhibiting drugs.

## CONFLICTS OF INTEREST

None to declare.

## AUTHOR CONTRIBUTIONS

**Krista Yordanova**: Conceptualization (Lead); Writing – original draft (Lead); Writing – review & editing (Lead). **Claudia Pfohler**: Conceptualization (Equal); Data curation (Equal); Project administration (Equal); Writing – original draft (Supporting); Writing – review & editing (Supporting). **Leonie Adam**: Conceptualization (Equal); Data curation (Equal); Resources (Equal). **Luca Schweitzer**: Conceptualization (Equal); Data curation (Equal); Formal analysis (Equal). **Catherine Bourg**: Conceptualization (Equal); Data curation (Equal); Formal analysis (Equal). **Thomas Vogt**: Project administration (Equal); Visualization (Equal); Writing – original draft (Supporting); Writing – review & editing (Supporting).

## ETHICS STATEMENT

Not applicable.

## Data Availability

Data available on request due to privacy/ethical restrictions.

## References

[1] Schilling B , Martens A , Geukes Foppen MH , Gebhardt C , Hassel JC , Rozeman EA , et al. First‐line therapy‐stratified survival in BRAF‐mutant melanoma: a retrospective multicenter analysis. Cancer Immunol Immunother. 2019;68(5):765–72. 10.1007/s00262-019-02311-1 30806748 PMC11028062

[2] Long GV , Hauschild A , Santinami M , Atkinson V , Mandala M , Chiarion‐Sileni V , et al. Adjuvant dabrafenib plus trametinib in stage III BRAF‐mutated melanoma. N Engl J Med. 2017;377(19):1813–23. 10.1056/nejmoa1708539 28891408

[3] Heinzerling L , Eigentler TK , Fluck M , Hassel JC , Heller‐Schenck D , Leipe J , et al. Tolerability of BRAF/MEK inhibitor combinations: adverse event evaluation and management. ESMO Open. 2019;4(3):e000491. 10.1136/esmoopen-2019-000491 31231568 PMC6555610

[4] Bellón T . Mechanisms of severe cutaneous adverse reactions: recent advances. Drug Saf. 2019;42(8):973–92. 10.1007/s40264-019-00825-2 31020549

[5] Cho YT , Chu CY . Treatments for severe cutaneous adverse reactions. J Immunol Res. 2017;2017:1503709. 10.1155/2017/1503709 29445753 PMC5763067

[6] Grünwald P , Mockenhaupt M , Panzer R , Emmert S . Erythema exsudativum multiforme, Stevens‐Johnson‐Syndrom/toxische epidermale Nekrolyse – Diagnostik und Therapie. JDDG J der Deutschen Dermatol Gesellschaft. 2020;18(6):547–53. 10.1111/ddg.14118_g 32519478

[7] Naqash AR , File DM , Ziemer CM , Whang YE , Landman P , Google PB , et al. Cutaneous adverse reactions in B‐RAF positive metastatic melanoma following sequential treatment with B‐RAF/MEK inhibitors and immune checkpoint blockade or vice versa. A single‐institutional case‐series 11 Medical and Health Sciences 1112 Oncology and Ca. J Immunother Cancer. 2019;7(1):1–9. 30621779 10.1186/s40425-018-0475-y PMC6323838

[8] Casagranda A , Suppa M , Dehavay F , del Marmol V . Overlapping DRESS and Stevens‐Johnson syndrome: case report and review of the literature. Case Rep Dermatol. 2017;9(2):1–7. 10.1159/000475802 PMC5465517 28611628

[9] Husain Z , Reddy BY , Schwartz RA . DRESS syndrome. Part I. Clinical perspectives. J Am Acad Dermatol. 2013;68(5):693. 10.1016/j.jaad.2013.01.033 23602182

[10] Schwartz RA , McDonough PH , Lee BW . Toxic epidermal necrolysis. Part I. Introduction, history, classification, clinical features, systemic manifestations, etiology, and immunopathogenesis. J Am Acad Dermatol Dermatol. 2013;69(2):173.e1–e13. quiz 185–186. 10.1016/j.jaad.2013.05.003 23866878

[11] Husain Z , Reddy BY , Schwartz RA . DRESS syndrome: Part II. Management and therapeutics. J Am Acad Dermatol. 2013;68(5):709. 10.1016/j.jaad.2013.01.032 23602183

[12] Paradisi A , Abeni D , Bergmo F , Ricci F , Didona D , Didona B . Etanercept therapy for toxic epidermal necrolysis. J Am Acad Dermatol. 2014;71(2):278–83. 10.1016/j.jaad.2014.04.044 24928706

[13] Leman RE , Chen L , Shi X , Rolimpandoei SP , Ling X , Su Y . Drug reaction with eosinophilia and systemic symptoms (DRESS) successfully treated with tumor necrosis factor‐α inhibitor. JAAD Case Rep. 2017;3(4):332–5. 10.1016/j.jdcr.2017.05.006 28752124 PMC5518151

[14] Scott LJE . A review of its use in autoimmune inflammatory diseases. Drugs. 2014;74(12):1379–410. 10.1007/s40265-014-0258-9 25034360

[15] Wang CW , Yang LY , Chen CB , Ho HC , Hung SI , Yang CH, et al. Randomized, controlled trial of TNF‐α antagonist in CTL‐mediated severe cutaneous adverse reactions. J Clin Invest. 2018;128(3):985–96. 10.1172/jci93349 29400697 PMC5824923

[16] Wojtkiewicz A , Wysocki M , Fortuna J , Chrupek M , Matczuk M , Koltan A . Beneficial and rapid effect of infliximab on the course of toxic epidermal necrolysis. Acta Derm Venereol. 2008;88(4):420–1. 18709327 10.2340/00015555-0462

[17] Paradisi A , Abeni D , Didona D , Ricci F , Canzona F , Didona B . A new case series on etanercept treatment for toxic epidermal necrolysis. Eur J Dermatol. 2020;30(5):561–8. 10.1684/ejd.2020.3883 33021477

[18] Zhang J , Lei Z , Xu C , Zhao J , Kang X . Current perspectives on severe drug eruption. Clin Rev Allergy Immunol. 2021;61(3):282–98. 10.1007/s12016-021-08859-0 34273058 PMC8286049

[19] Ros J , Muñoz‐Couselo E . DRESS syndrome due to vemurafenib treatment: switching BRAF inhibitor to solve a big problem. BMJ Case Rep. 2018;2018:bcr2018224379. 10.1136/bcr-2018-224379 PMC6169641 30275021

